# Predictive value of echocardiographic parameter of diastolic dysfunction (Ea/Aa) combined with electrocardiographic P-wave dispersion for the detection of early recurrence of atrial fibrillation after radiofrequency catheter ablation

**DOI:** 10.3389/fcvm.2025.1585919

**Published:** 2025-08-28

**Authors:** Xuebin Ling, Jiaqi Li, Julan Wu, Yimin He, Minfang Wu, Yanjun Hou, Tianfa Li, Zhenling Wan, Chufen Lin

**Affiliations:** ^1^ Key Laboratory of Emergency and Trauma of Ministry of Education, Department of Cardiovascular Medicine, The First Affiliated Hospital of Hainan Medical University, Haikou, China; ^2^ Hainan Provincial Key Laboratory for Tropical Cardiovascular Diseases Research, Engineering Research Center for Hainan Biological Sample Resources of Major Diseases, The First Affiliated Hospital of Hainan Medical University, Haikou, China; ^3^ Department of Pathology, Hainan Women and Children Medical Center, Hainan Medical University, Haikou, China; ^4^ Department of Health Management, The Affiliated Haikou Hospital of Xiangya Medical College Central South University, Haikou, China

**Keywords:** Ea/Aa, P-wave dispersion, atrial fibrillation, early recurrence, radiofrequency catheter ablation

## Abstract

**Objective:**

To explore the predictive value of echocardiographic parameters of left-ventricular diastolic function combined with electrocardiographic P-wave dispersion (PWD) for early recurrence of atrial fibrillation (ERAF) after radiofrequency catheter ablation (RFCA).

**Methods:**

A total of 145 patients with atrial fibrillation who underwent their first RFCA at the Department of Cardiology, First Affiliated Hospital of Hainan Medical University, between January 2021 and December 2024, were enrolled in the study. The patients were followed up for 3 months after RFCA and divided into two groups, namely those with early recurrence and those without early recurrence. Within 2 h after the procedure, 12-lead electrocardiogram (ECGs) was recorded. The maximum P-wave duration (Pmax), minimum P-wave duration (Pmin), and PWD (the difference between Pmax and Pmin) were measured. Additionally, left-ventricular diastolic function parameters were obtained via echocardiography.

**Results:**

ECG monitoring revealed ERAF in 45 patients (31.03%) at 3-month follow-up after RFCA. Spearman correlation analysis showed that ERAF positively correlated with PWD (*r* = 0.68, *P* < 0.001) and negatively correlated with Ea/Aa ratio (*r* = −0.49, *P* < 0.001). Under the combination of Ea/Aa < 1 and PWD, the area under the receiver operating characteristic (ROC) curve for the combined prediction increased to 0.95 [95% confidence interval (CI) = 0.91–0.98], with a Youden index of 0.73 (sensitivity 83.83%, specificity 88.88%). The incidence of ERAF was significantly higher in the group with Ea/Aa ratio <1 than in the group with Ea/Aa ratio ≥1 (66.07 vs. 9.09%, *P* < 0.001). The incidence of ERAF was significantly higher in the group with PWD ≥ 29.5 ms than in the group with PWD < 29.5 ms (65.08 vs. 4.88%, *P* < 0.001). Ea/Aa ratio and PWD were independently associated with ERAF [for Ea/Aa ratio, adjusted hazard ratio (HR) = 13.48, 95% CI = 3.49–52.05, *P* < 0.001; for PWD, adjusted HR = 0.23, 95% CI = 0.08–0.62, *P* = 0.037, respectively].

**Conclusion:**

Left-ventricular diastolic dysfunction parameters and PWD can effectively predict ERAF after RCAF. Ea/Aa < 1 and PWD ≥ 29.5 ms are strong and independent predictors of ERAF.

## Introduction

Atrial fibrillation (AF) can lead to left-atrial remodeling, causing myocardial fibrosis, left-atrial enlargement, and reduced left-ventricular contractility, which increases the risk of cardiovascular complications and death (1). Catheter radiofrequency ablation (RFCA) is currently the first-line treatment for AF, showing significant efficacy in maintaining sinus rhythm, relieving symptoms, and improving patients’ quality of life (2). Results from multiple clinical trials (3, 4) have shown that cardiac rhythm control through RFCA can improve cardiac function in patients with AF, alleviate symptoms and exercise tolerance, and reduce the risk of heart failure and mortality. However, AF recurrence remains an issue, with more than 50% (5) of patients experiencing early recurrence of AF (ERAF). ERAF refers to the occurrence of rapid atrial arrhythmias lasting more than 30 s within 3 months after the procedure, which is associated with an increased risk of late recurrence (6). Some ERAF patients have a poor prognosis even with secondary RFCA treatment, and may experience hemodynamic instability, renal embolism, or even heart failure. Therefore, finding predictive indicators for ERAF is of great significance for reducing the AF recurrence rate after RFCA.

The atrial structural and electrical remodeling–induced atrial conduction abnormalities are the most important pathophysiological mechanisms underlying the onset of AF. Our previous study (7) showed that increased left-atrial pressure is an important risk factor for early recurrence. Recent studies (4) have shown that 3 months after RFCA, left-atrial function predicts atrial arrhythmia recurrence following ablation of long-standing persistent AF. To assess left-ventricular (LV) diastolic function, tissue Doppler imaging was applied to determine Ea/Aa, the ratio between the velocity of the early (Ea) and late (Aa) apical movement of the mitral annulus, and conventional Doppler imaging was used to measure E/A, the ratio between early mitral inflow velocity (E) and late mitral inflow velocity (A). Recent studies have shown that LV diastolic dysfunction parameters, such as E/A < 1 and Ea/Aa < 1, can indirectly reflect elevated left-atrial pressure (8). The P-wave dispersion (PWD), calculated as the difference between the longest P-wave duration (Pmax) and the shortest P-wave duration (Pmin) measured from a 12-lead electrocardiogram (ECG), is one of the parameters representing atrial conduction heterogeneity and is associated with AF recurrence (7, 9). The study was the first to explore the combined predictive efficacy of left-atrial pressure remodeling indicators (E/A < 1, Ea/Aa < 1) and left-atrial electrical remodeling indicators (PWD) in predicting ERAF after RFCA, aiming to provide theoretical support for clinical application.

## Materials and methods

### Study population

The present study consecutively enrolled 145 hospitalized patients with AF at the First Affiliated Hospital of Hainan Medical University from January 2021 to December 2024. All of the patients underwent their first RFCA, and sinus rhythm was restored following the procedure. Among the participants, 90 were male and 55 were female, with an average age of 60.6 ± 11.8 years. ERAF was defined as AF episodes lasting longer than 30 s within 3 months after RFCA, detected by ECG or Holter monitoring.

Inclusion criteria were as follows: AF patients scheduled for RFCA, aged <80 years; left-atrial diameter <55 mm; and transesophageal echocardiography showing no thrombus in the left-atrial appendage or left atrium, or evidence of spontaneous echocardiographic contrast. Exclusion criteria were as follows: acute or end-stage liver or kidney diseases, severe chronic obstructive pulmonary disease, acute coronary syndrome, malignancies, severe infections, clinically and echocardiographically confirmed congenital heart disease, valvular heart disease, or previous valve replacement; left-atrial diameter >55 mm and left-ventricular ejection fraction <45%; clinical hyperthyroidism; previous AF ablation or pacemaker implantation; poor ECG quality affecting measurement; incomplete data; AF episodes during echocardiographic examination (precluding the measurement of E, A, Ea, or Aa values); and inability to follow up. The study was approved by the Ethics Committee of the First Affiliated Hospital of Hainan Medical University (Ethics approval number: 2022-129), and all patients (and/or their families) were fully informed of the research and voluntarily agreed to participate.

### Electrophysiological examination and RFCA procedures

Before surgery, the patients received at least 1 month of warfarin anticoagulation therapy, and atrial thrombus was excluded within 24 h prior to RFCA using transesophageal echocardiography and pulmonary vein computed tomography angiography (CTA). During the procedure, heparin was administered based on body weight at 1,000 U/kg to maintain the activated clotting time (ACT) between 300 and 350 s. After successful puncture of the interatrial septum, Pentaray (Biosense Webster) was used under the guidance of the Carto 3 electroanatomical mapping system (Biosense Webster). A star-shaped electrode was inserted into the left atrium for mapping and modeling. After modeling, a saline infusion ablation catheter STSF (Biosense Webster) was introduced into the left atrium for pulmonary vein potential localization. A power mode was selected for paroxysmal AF (ParAF) ablation, and the ablation index (AI) was used to guide the circumferential pulmonary vein ablation. The parameters were set as follows: posterior wall power 40 W, AI = 380; superior and inferior walls power 45 W, AI = 400; and anterior wall power 45 W, AI = 450. The left and right pulmonary veins were isolated along the atrial vestibular potentials. For persistent AF (PerAF), in addition to the ParAF ablation lines, the left atrial roof line (power 45 W, AI = 400) and left-atrial posterior wall line (power 45 W, AI = 400) were also ablated. Some patients underwent mitral isthmus line ablation (power 50 W, AI = 500), tricuspid isthmus line ablation (power 40 W, AI = 400), and superior vena cava isolation (power 40 W, AI = 400) for atrial flutter or fibrillation originating from the superior vena cava. Sinus rhythm pacing stimulation was performed for corresponding site stimulation mapping to confirm whether the left-atrial roof line, floor line, superior vena cava line, mitral isthmus line, and tricuspid isthmus line had been successfully blocked. Successful RFCA was defined as closure at the pulmonary vein exit, closure at the left-atrium entry, and no atrial arrhythmia induced within 30 min after pulmonary vein isolation, with bidirectional block at the pulmonary vein–left atrium junction. Postoperatively, the patients were given oral warfarin and amiodarone. The medication doses were adjusted based on INR, liver and kidney function, and heart rate, and were continued for 3 months.

### Echocardiographic examination

**Figure 1:**
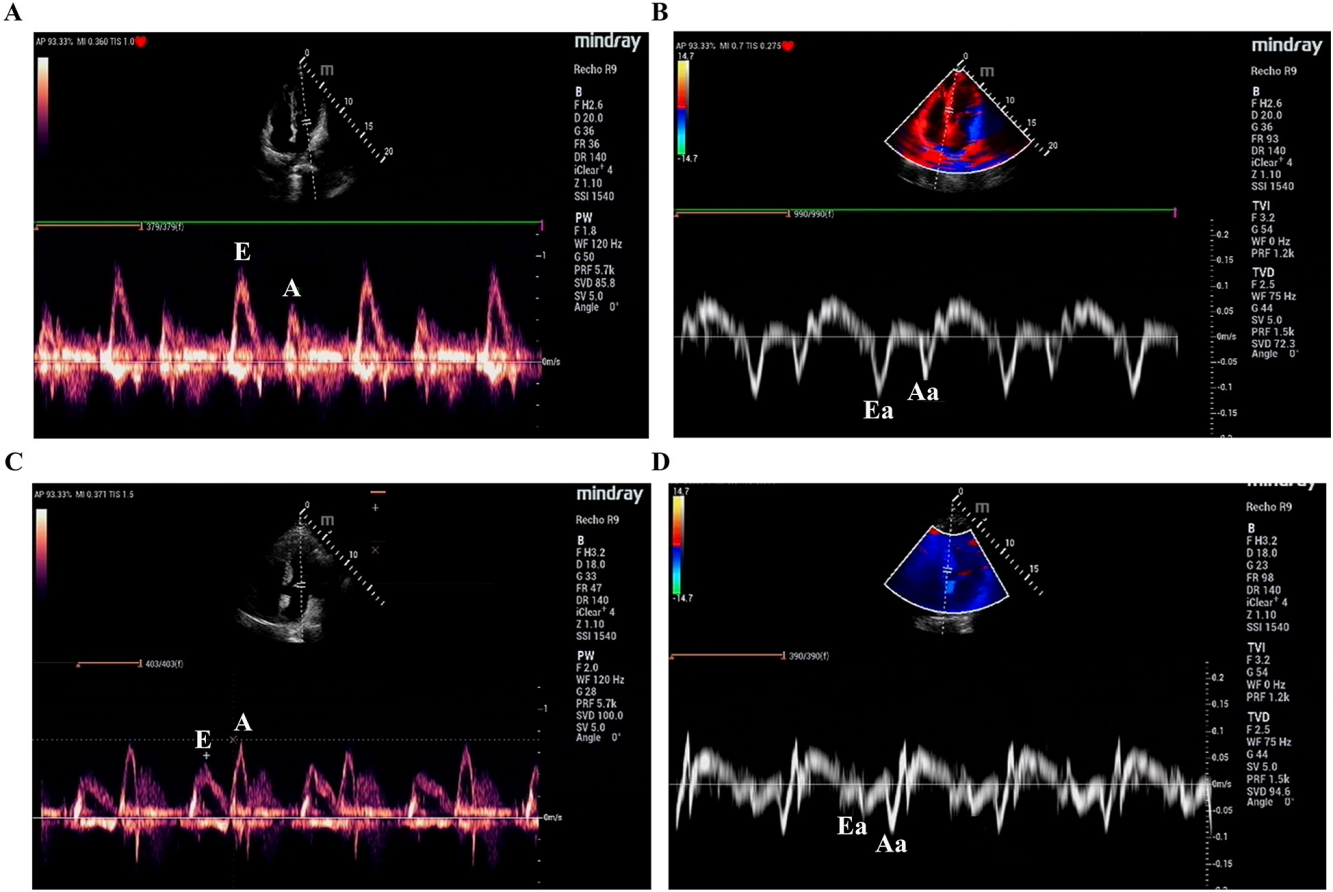
The pulsed Doppler **(A,C)**/tissue Doppler **(B,D)** imaging. Early mitral inflow velocity (E); Late mitral inflow velocity (A); Eearly diastolic myocardial relaxation velocity (Ea); Late diastolic velocity (Aa).

In the apical four-chamber view, the pulsed Doppler/tissue Doppler imaging (PW/TDI) mode was used to measure the intracardiac blood flow velocity generated by the pressure gradient between the left atrium and the left ventricle. These measurements were obtained from the apical four-chamber view, with the early diastolic filling (E wave) representing the left-ventricular early filling and the late diastolic filling (A wave) representing atrial contraction. In cases of normal diastolic function, the early ventricular filling component is significantly higher than the atrial contraction component, i.e., E/A > 1. TDI measures the velocity of myocardial length changes, with the sample volume located in the ventricular muscle near the mitral annulus, between the free wall and the side wall insertion points of the mitral valve leaflets in the apical four-chamber view. Early diastolic myocardial relaxation velocity (Ea) corresponds to the circumferential movement toward the apex, while late diastolic velocity (Aa) corresponds to atrial contraction (Figure 1). In healthy individuals, Ea/Aa is >1. All acquired echocardiographic images were reviewed by at least two experienced echocardiologists, and a consensus was reached after discussion. The measurements for each parameter were averaged over five cardiac cycles. The main parameters for cardiac examination included the following: left-atrial diameter (mm), left-ventricular end-diastolic diameter (LVEDD, mm), left-ventricular end-systolic diameter (LVESD, mm), left-ventricular ejection fraction (%), maximum mitral flow velocity during early diastole (E), maximum mitral flow velocity during atrial contraction (A), E/A ratio, Ea, Aa, and Ea/Aa ratio.

### Measurement of ECG parameters

**Figure 2:**
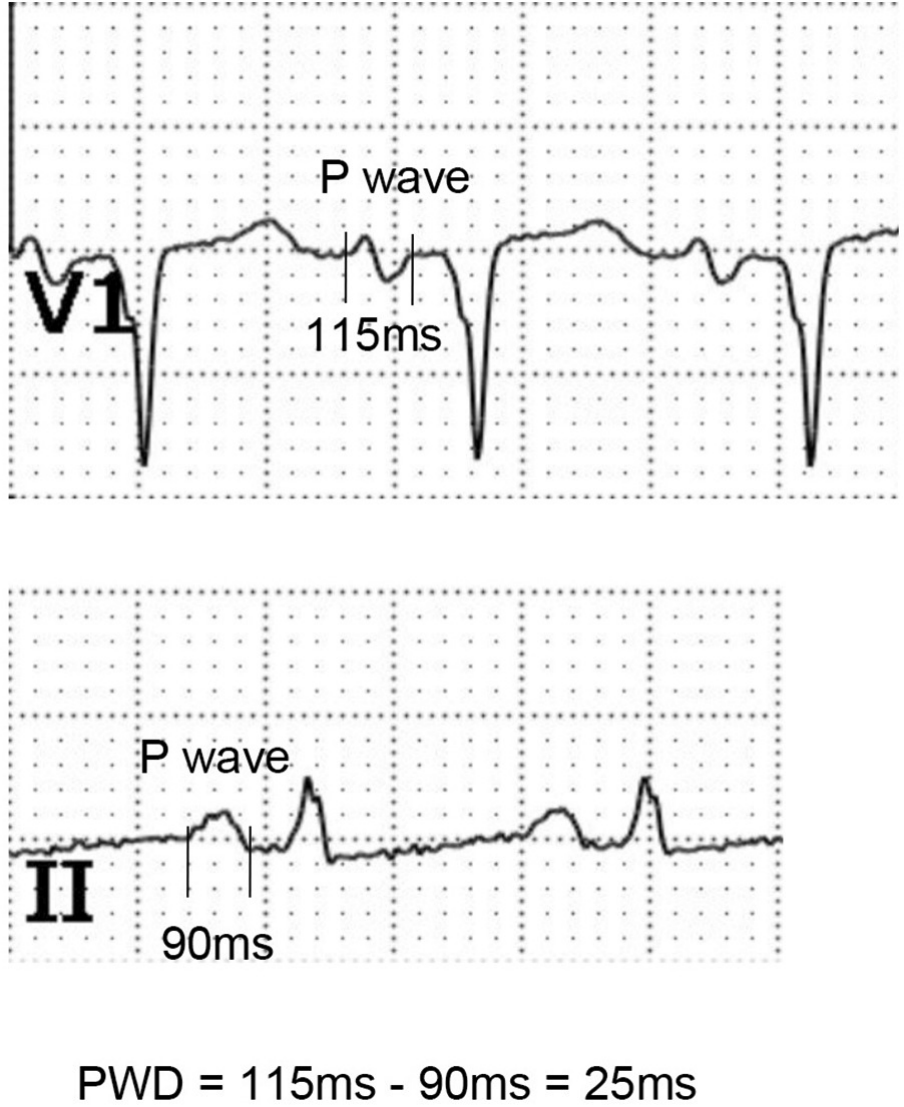
Schematic diagram of P-wave dispersion (PWD) measurement.

The AF patients who underwent RFCA were subjected to standard 12-lead surface ECG with a paper speed of 25 mm/s. The P-wave onset was defined as the junction between the isoelectric line and the beginning of the P wave, while the P-wave termination was defined as the junction between the isoelectric line and the end of the P wave. P-wave dispersion (PWD), a marker of prolonged atrial conduction time, was calculated as the difference between the longest and shortest P-wave durations obtained from the 12-lead ECG after restoring sinus rhythm (Figure 2). The mean P-wave duration difference (mPWD) was defined as the average of the maximum and minimum P-wave durations. Post-ablation follow-up included daily ECG checks during hospitalization, as well as routine ECG examinations at 1, 3, 6, and 12 months after discharge. If any patient experienced symptoms such as chest tightness and palpitations, an ECG was performed immediately. The follow-up method included regular outpatient follow-up visits, with the recurrence status assessed at 3 months after ablation. The ECG recurrence criteria were defined as episodes of AF, atrial tachycardia, or atrial flutter recorded on the surface ECG when the patient experienced symptoms of tachycardia. These data were independently measured by two ECG diagnostic physicians using the Nalong system (Nalong Technology, China).

### Statistical analysis

Data were processed using SPSS 27.0 statistical software. For normally distributed continuous variables, the mean ± standard deviation (SD) was used, and comparisons between two groups were performed using the Student's *t* test. For non-normally distributed continuous variables, the median (interquartile range) was used, and comparisons between two groups were conducted using nonparametric tests. Categorical data were expressed as percentages (%), and comparisons between two groups were made using the *χ*^2^ test. Factors influencing ERAF were analyzed using multivariate logistic regression analysis. Correlations were assessed using the Spearman correlation coefficient, with a significance level set at *α* = 0.05. The predictive efficacy for ERAF was evaluated using receiver operating characteristic (ROC) curve analysis. Kaplan–Meier analysis with the log-rank test was used to compare the ERAF rates between the two categories based on E/A, Ea/Aa, and PWD. Variables with *P* < 0.10 between the recurrence group and the non-recurrence group were included in the Cox multivariate regression model (Enter method) to analyze the predictors of ERAF. A two-sided *P* value lower than 0.05 was considered statistically significant.

## Results

### Differences in clinical characteristics between the patients with ERAF and those without

**Figure 3:**
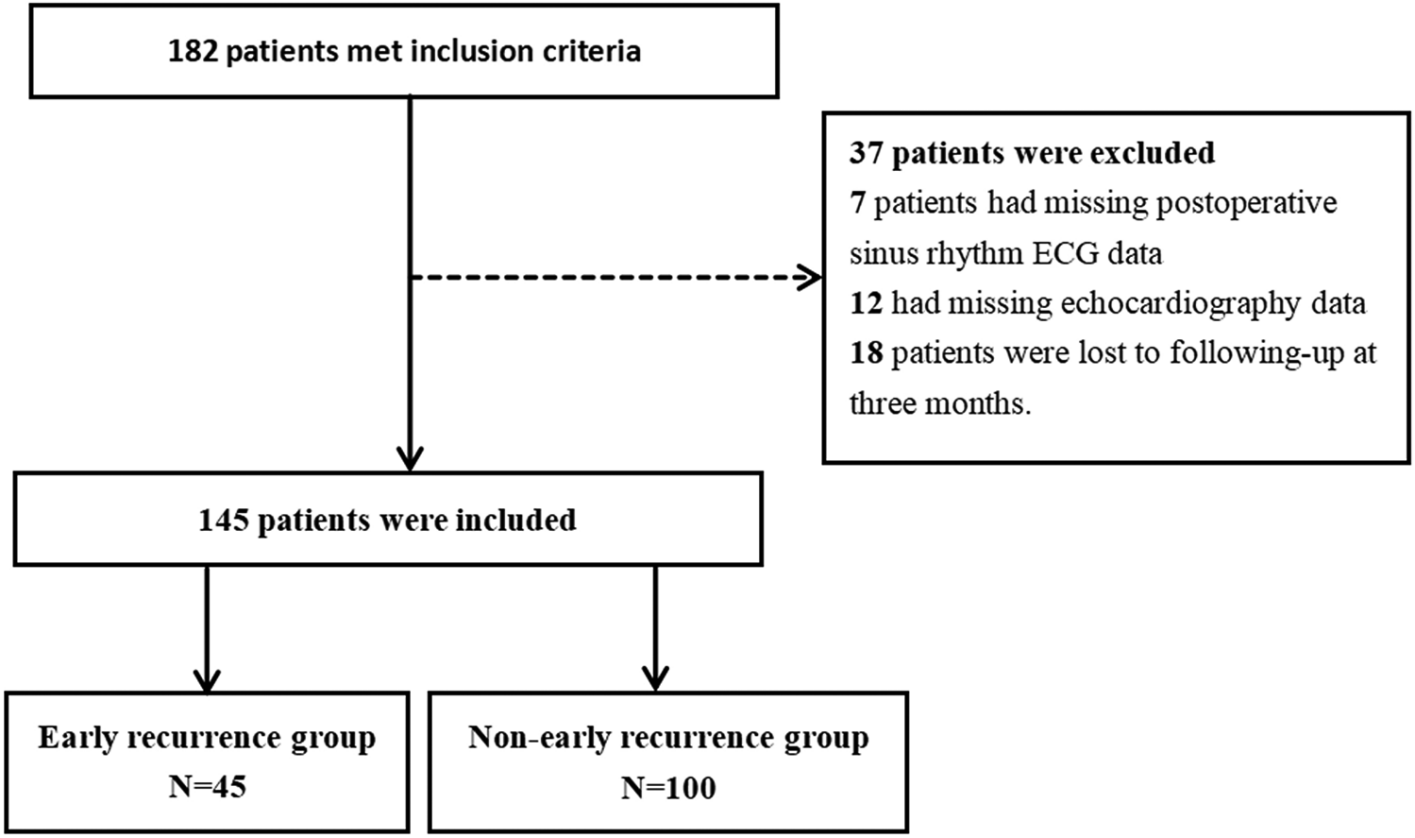
Selection process of research subjects.

This study included a total of 145 AF who underwent RFCA at the First Affiliated Hospital of Hainan Medical University from January 2022 to June 2024. The patients were followed up for 90 days, during which 45 patients experienced ERAF, while 100 patients showed no recurrence, constituting the final study population (Figure 3). The average age of the recurrence group was 61.4 ± 13.0 years, with 24 males (53.3%), and the average age of the non-recurrence group was also 61.4 ± 13.0 years, with 24 males (53.3%). Statistical analysis of the general clinical data of the two groups revealed no significant differences in age, gender, body mass index (BMI), heart rate, AF type, smoking, diabetes, coronary heart disease, or laboratory indicators (*P* > 0.05). However, there were significant differences in blood pressure levels, AF duration, E/A ratio, E/A < 1, Ea/Aa, Ea/Aa < 1, and PWD between the two groups (*P* < 0.05), as shown in Table 1.

**Table 1:** Baseline characteristics of the patients with ERAF and those without.

Variable	ERAF (*n* = 45)	No ERAF (*n* = 100)	F/*χ*^2^/Z	*P* value
Age, years	61.4 ± 13	60.3 ± 11.1	0.2	0.61
Male, *n* (%)	24 (53.3)	66 (66)	2.1	0.15
BMI, kg/m^2^	24.4 ± 2.4	24.1 ± 2.9	1.133	0.332
Systolic blood pressure, mm Hg	131.3 ± 20.9	119.5 ± 32.5	6.8	0.01
Diastolic blood pressure, mm Hg	82.6 ± 13.3	75.2 ± 20.3	4.9	0.03
Heart rate, bpm	76. ± 17.6	78 ± 26.2	0.2	0.65
ERAF, days	33.8	0	139	<0.001
Paroxysmal AF, *n* (%)	17 (37.8)	44 (44)	0.49	0.48
Smoker, *n* (%)	10 (22.2)	22 (22.0)	0.001	0.98
Diabetes mellitus, *n* (%)	8 (17.8)	14 (14.0)	0.99	0.61
Coronary artery disease, *n* (%)	6 (13.3)	19 (19.0)	0.7	0.4
Warfarin/rivaroxaban, *n* (%)	39 (86.7)	94 (94.0)	6.1	0.15
Amiodarone, *n* (%)	36 (80.0)	80 (80.0)	<0.01	1
Beta blocker, *n* (%)	7 (15.6)	16 (16.0)	0.005	0.95
WBC, ×10^9 ^/L	6.5 ± 1.9	6.7 ± 1.8	0.1	0.73
HGB, g/L	132.5 ± 31.6	139.8 ± 15.9	3.5	0.06
PLT, ×10^9 ^/L	212.6 ± 62.8	228.8 ± 57.4	2.3	0.13
PT-INR	1 ± 0.09	1 ± 0.2	0.3	0.6
CREA, μmol/L	70.4 ± 19.4	76.3 ± 18.2	3.1	0.08
TSH, mIU/L	1.9 ± 1.5	2 ± 1.7	0.06	0.81
FT3, pmol/L	4.6 ± 0.8	4.9 ± 1.2	1.9	0.17
FT4, pmol/L	15.6 ± 3.1	15.9 ± 3.7	0.2	0.73
LDL-C, mmol/L	2.8 ± 0.9	2.9 ± 1.3	0.2	0.63
AF duration, months	6 [3, 22.5]	6 [2, 13.5]	−2.9	0.004
PWD, ms	46.3 ± 14.4	21.4 ± 10.5	137.8	<0.001
mPWD, ms	95.6 ± 17	98.1 ± 21.5	0.5	0.49
Left atrium, mm	42.3 ± 18	41.4 ± 6.8	0.6	0.44
Right atrium, mm	36.3 ± 5.5	35.2 ± 4.9	1.2	0.28
Left ventricle, mm	48.2 ± 5.5	48.9 ± 5.4	0.4	0.52
Right ventricle, mm	22.2 ± 4.5	21.6 ± 2.9	0.7	0.42
LVEF, %	62.66 ± 8.68	62.75 ± 9.90	0.56	0.45
TRV, m/s	22.2 ± 4.7	21.6 ± 2.11	1.6	0.21
E/A	0.75 [0.65, 0.87]	1.15 [0.85, 1.34]	−0.22	0.83
E/A < 1, *n* (%)	22 (48.9)	22 (22.2)	10.36	0.001
Ea/Aa	0.76 ± 0.25	1.15 ± 0.38	39.2	<0.001
Ea/Aa < 1, *n* (%)	37 (82.2)	19 (19.2)	51.72	<0.001
CHA2DS2-Vasc score	2.5 ± 1.4	2.6 ± 1.6	0.05	0.82
HASBLED score	1.1 ± 0.8	1.1 ± 0.9	0.01	0.91

ERAF, early recurrence of atrial fibrillation; PWD, P-wave dispersion; E/A, ratio between early mitral inflow velocity (E) and late mitral inflow velocity (A); Ea/Aa, ratio between early diastolic myocardial relaxation velocity (Ea) and late diastolic velocity (Aa); BMI, body mass index; LVEF, left-ventricular ejection fraction; TRV, tricuspid regurgitation velocity.

### Correlation analysis between left-ventricular diastolic function parameters and PWD

**Figure 4:**
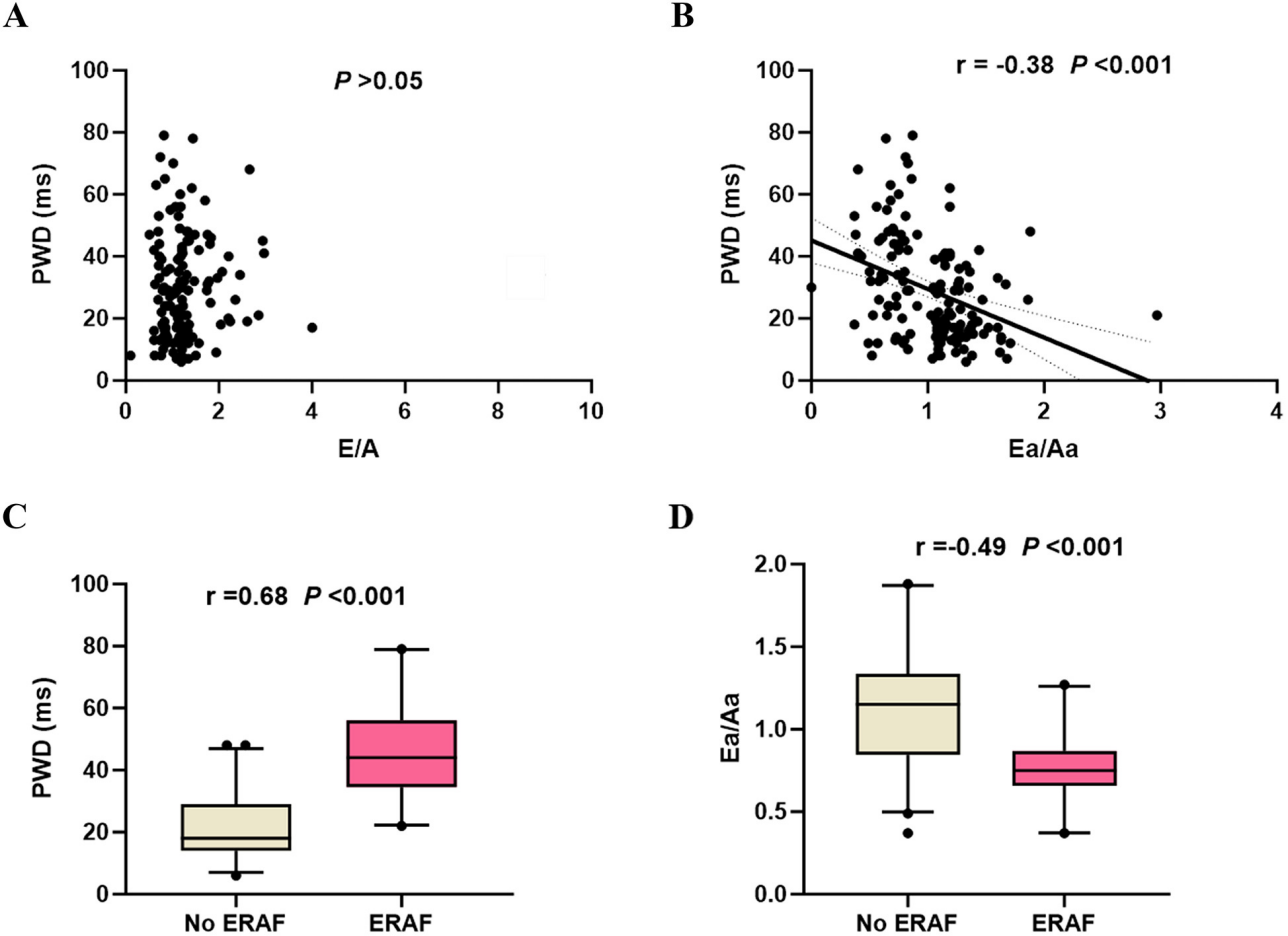
Spearman correlation analysis. **(A)** Scatter plot of the correlation between the E/A ratio and PWD; **(B)** Scatter plot of the correlation between the Ea/Aa ratio and PWD; **(C)** Box plot showing the correlation between ERAF and PWD values; **(D)** Box plot showing the correlation between ERAF and Ea/Aa values. ERAF, early recurrence of atrial fibrillation; PWD, P-wave dispersion; E/A, ratio between early mitral inflow velocity (E) and late mitral inflow velocity (A); Ea/Aa, ratio between early diastolic myocardial relaxation velocity (Ea) and late diastolic velocity (Aa).

After RFCA and restoration of sinus rhythm in AF patients, both ECG and echocardiographic examinations were performed. Spearman correlation analysis demonstrated that PWD had no correlation with the E/A ratio, a parameter of left-ventricular diastolic dysfunction (*P* > 0.05), but showed negative correlation with the Ea/Aa ratio (*r* = −0.38, *P* < 0.001). Further analysis of the recurrence and non-recurrence groups with respect to categorical variables and PWD and Ea/Aa values showed that ERAF was positively associated with PWD (*r* = 0.68, *P* < 0.001) and negatively associated with Ea/Aa (*r* = −0.49, *P* < 0.001), suggesting that increased PWD and reduced Ea/Aa are closely associated with ERAF after ablation, as shown in Figure 4.

### Prediction of ERAF after RFCA using left-ventricular diastolic dysfunction parameters and PWD

**Figure 5:**
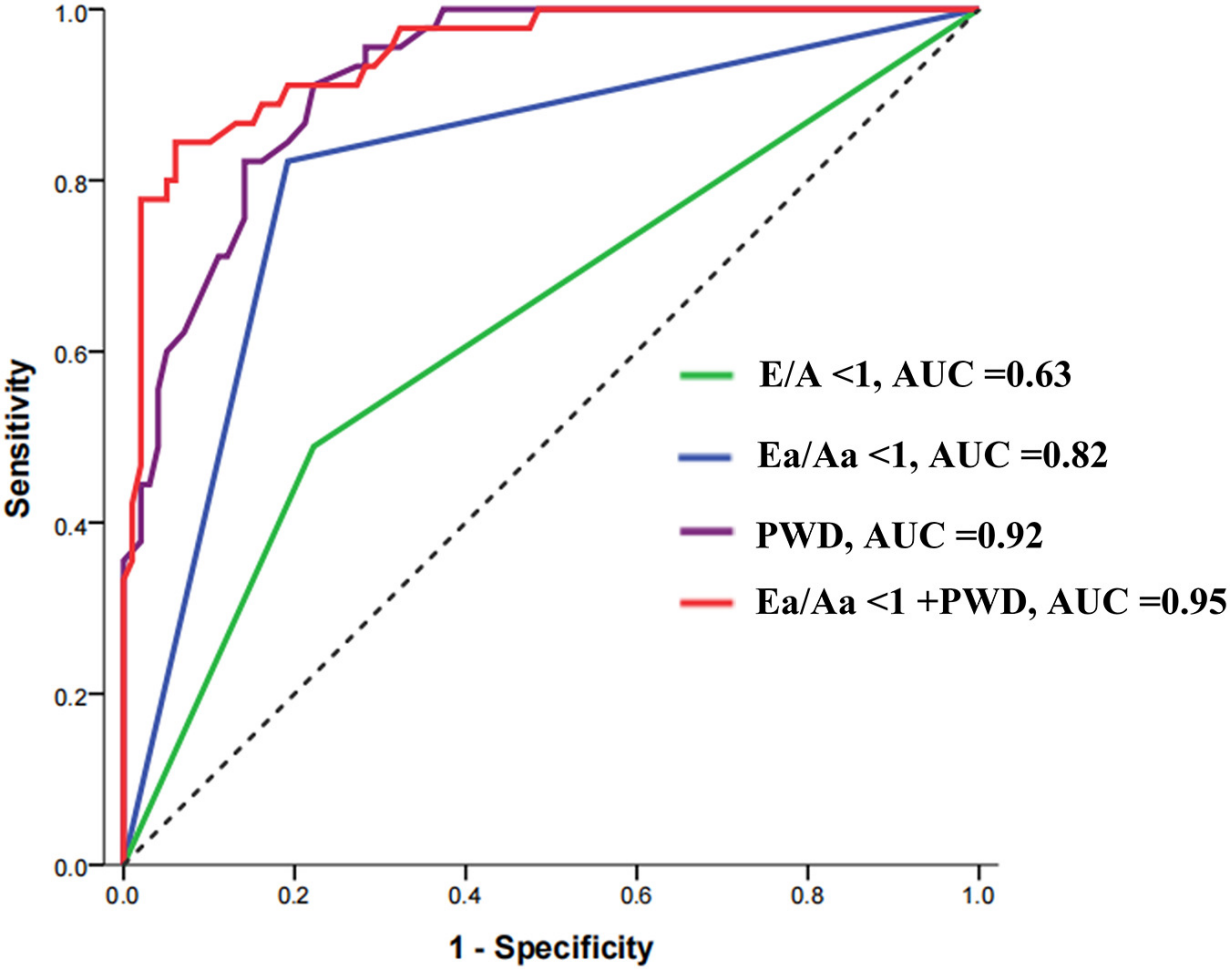
Receiver operating characteristic curve for the predictive value of PWD, E/A, and Ea/Aa ratios in early recurrence after radiofrequency catheter ablation in patients with atrial fibrillation. PWD, P-wave dispersion; E/A, ratio between early mitral inflow velocity (E) and late mitral inflow velocity (A); Ea/Aa, ratio between early diastolic myocardial relaxation velocity (Ea) and late diastolic velocity (Aa); AUC, area under the curve.

ROC curve analysis revealed the following predictive efficacy for ERAF after RFCA. E/A < 1 had an area under the ROC curve (AUC) of 0.63 [95% confidence interval (CI) = 0.53–0.74] for predicting ERAF, while Ea/Aa < 1 had an AUC of 0.82 (95% CI = 0.74–0.89) for predicting ERAF. PWD had an AUC of 0.92 (95% CI = 0.88–0.96) for predicting ERAF, with a Youden index of 0.69 and an optimal cutoff value of 29.5 ms. The sensitivity was 77.77%, and the specificity was 91.11%. When Ea/Aa < 1 and PWD were combined in a binary logistic regression model, the AUC for the combined prediction increased to 0.95 (95% CI = 0.91–0.98), with a Youden index of 0.73 (sensitivity 83.83%, specificity 88.88%), which was superior to the prediction accuracy of either parameter alone (Figure 5).

### Kaplan–Meier survival curve analysis for ERAF

**Figure 6:**
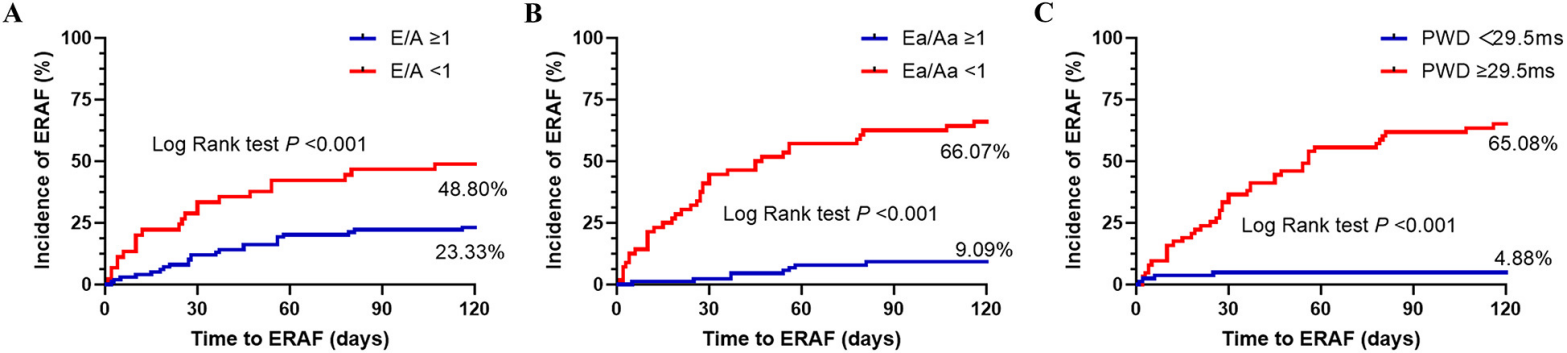
Kaplan–Meier curve of ERAF after RFCA in different groups based on the E/A ratio cutoff value of 1 **(A)**, Ea/Aa ratio cutoff value of 1 **(B)**, and PWD cutoff value of 29.5 ms **(C)** at 3-month follow-up. ERAF, early recurrence of atrial fibrillation; E/A, ratio between early mitral inflow velocity (E) and late mitral inflow velocity (A); Ea/Aa, ratio between early diastolic myocardial relaxation velocity (Ea) and late diastolic velocity (Aa).

The ERAF rate was 23.33% in the group with the E/A ratio ≥1 and 48.80% in the group with the E/A ratio <1. The difference between the two groups was statistically significant (*P* < 0.001). The incidence of ERAF was significantly higher in the group with the Ea/Aa ratio <1 compared with the group with the Ea/Aa ratio ≥1 (66.07 vs., 9.09%, *P* < 0.001). Based on the optimal cutoff value of PWD (29.5 ms), the PWD values were divided into two groups, namely ≥29.5 ms and <29.5 ms. The incidence of ERAF was significantly higher in the group with PWD ≥ 29.5 ms than in the group with PWD < 29.5 ms (65.08% vs. 4.88%, *P* < 0.001) (Figure 6).

### Multivariate analysis of ERAF

Using PWD ≥ 29.5, E/A ≥ 1, and Ea/Aa ≥ 1 as categorical variables, univariate and multivariate Cox regression analyses were conducted on the factors influencing ERAF, including age, gender, blood pressure levels, AF duration, PWD, E/A, and Ea/Aa. After adjusting for gender, systolic blood pressure, diastolic blood pressure, and AF duration, multivariate Cox regression analysis showed that PWD ≥ 29.5 ms was a risk factor for ERAF after RFCA, with a hazard ratio (HR) of 13.48 (95% CI = 3.49–52.05, *P* < 0.001). In contrast, Ea/Aa ≥ 1 was identified as a protective factor for ERAF after RFCA, with an HR for late recurrence of 0.23 (95% CI = 0.08–0.62, *P* = 0.037). Other variables in this model were not independent risk factors for ERAF, as shown in Table 2.

**Table 2:** Multivariate analysis to identify the individual risk factors associated with ERAF after RFCA.

Variable	Univariate		Multivariate	
	HR (95% CI)	*P* value	HR (95% CI)	*P* value
Age, years	1.01 (0.98, 1.04)	0.51	1.00 (0.97, 1.03)	0.94
Male, *n* (%)	0.63 (0.35, 1.13)	0.12	0.89 (0.48, 1.66)	0.71
SBP, mm Hg	1.02 (1.00, 1.03)	0.01	1.01 (0.99, 1.03)	0.7
DBP, mm Hg	1.02 (1.00, 1.03)	0.04	0.99 (0.95, 1.02)	0.86
AF duration, months	1.03 (1.02, 1.05)	<0.001	1.00 (0.98, 1.02)	0.91
PWD, ms	1.06 (1.04, 1.07)	<0.001	1.00 (0.98, 1.03)	0.95
PWD ≥ 29.5 ms	20.18 (7.20, 56.58)	<0.001	13.48 (3.49, 52.05)	<0.001
E/A	1.24 (0.97, 1.60)	0.085	1.16 (0.90, 1.50)	0.25
E/A ≥ 1, *n* (%)	0.37 (0.21, 0.67)	0.001	0.47 (0.22, 1.00)	0.051
Ea/Aa	0.08 (0.03, 0.19)	<0.001	0.53 (0.13, 2.11)	0.37
Ea/Aa ≥ 1, *n* (%)	0.09 (0.04, 0.19)	<0.001	0.23 (0.08, 0.62)	0.037

## Discussion

AF is the most common arrhythmia, affecting 2% of the global population, with over 34 million patients worldwide, and its prevalence is expected to double by 2050 (10). Patients with AF lose the active contraction and pumping function of the left atrium, and atrial function during the catheterization and storage phases declines (11). Cardiac output decreases by 20%, left-atrial pressure increases, and left-atrial volume and fibrosis increase, which triggers left-atrial remodeling and ectopic electrical activity. The frequency of AF attacks increases with age, and ParAF easily converts to PerAF, which can significantly increase the risk of thromboembolic events and congestive heart failure. Although the pathogenesis of AF has not yet been clarified, it has been confirmed that the rapid ectopic pacemakers that cause AF are mostly located in the pulmonary veins and left-atrial muscle sleeves. Therefore, circumferential pulmonary vein isolation has become the cornerstone strategy for the treatment of AF (2). However, there is still a certain recurrence rate after AF ablation. Previous studies have shown that the recurrence of AF may be related to atrial structural remodeling (12), atrial electrical remodeling (13), atrial fibrosis (14), increased atrial pressure (15), and increased blood pressure causing atrial overload (16). Magni et al. (17) found in a follow-up study that a higher degree of left-ventricular fibrosis in AF patients with RFCA was associated with a greater risk of postoperative recurrence. The velocity of left-atrium appendage (LAA) wall motion during AF is a potential marker of mechanical remodeling, and Paweł et al. reported that the velocity of LAA wall motion during AF predicted the success of electrical cardioversion and long-term sinus rhythm maintenance (18). Diastolic dysfunction of the left ventricle is an important risk factor for cardiovascular disease, which can also lead to compensatory enlargement of the left-atrial pressure and volume in patients with AF; this in turn causes left-atrial tissue and electrical remodeling, negatively impacting the function of the left atrium. This increases the likelihood of conduction disturbances or delays within the atrium, thereby raising the risk of early recurrence after RFCA for AF (19). There are several potential pathological mechanisms. Importantly, left-ventricular diastolic dysfunction leads to stretching and enlargement of the left atrium and pulmonary veins, which facilitates the onset of AF. Once AF occurs, it can induce both electrical and anatomical remodeling of the heart (20). In addition, when left-ventricular diastolic function is impaired, compensatory enlargement of the left-atrial pressure and volume occurs. As progressive electrical and tissue remodeling of the left atrium continues, it leads to pulmonary vein dilation and congestion, which further facilitates the occurrence of AF (21). After RFCA, patients are in a “window period” within the first 3 months, during which the heart tissue is healing, left-atrial pressure may fluctuate, autonomic nervous regulation changes, and inflammatory stimuli may affect atrial electrical activity. These factors can lead to ERAF, significantly reducing the benefits of the procedure and resulting in poor prognosis. Mohanty et al. (22) reported that early recurrence occurring in the second or third month—rather than the first month—following pulsed field ablation (PFA) was more strongly associated with an increased risk of late recurrence. Based on these findings, the blanking period was redefined as the first month after PFA. In our study, ERAF was observed in 45 patients (31%): 24 cases occurred in the first month, 16 in the second month, and 5 in the third month. In light of this, we further investigated the predictive value of Ea/Aa and P-wave dispersion (PWD) specifically for ERAF within the first month. The results demonstrated that Ea/Aa < 1 yielded an AUC of 0.80, while PWD showed an AUC of 0.86 for predicting ERAF. When Ea/Aa < 1 and PWD were combined in a binary logistic regression model, the AUC improved to 0.89, indicating enhanced predictive performance (Supplementary Figure S1). These findings suggest that Ea/Aa < 1 and PWD retain strong predictive value for ERAF even under a shortened blanking period, and may serve as useful markers for early risk stratification. Therefore, it is essential to identify patients at high risk of ERAF in order to implement targeted preventive and therapeutic measures in a timely manner.

Many predictors of ERAF have been identified, including older age, structural heart disease, longer AF duration, nonparoxysmal AF type, and larger size and volume of the left atrium (23, 24). Biomarkers of cardiac remodeling, such as higher angiopoietin-2 and interleukin-6 levels, are associated with recurrence after RFCA (25). However, the aforementioned indicators lack specificity, which limits their clinical utility in predicting ERAF. Increased atrial pressure leading to elevated wall stress plays a pivotal role in the development and progression of both electrical and structural remodeling of the atria. According to the guidelines established by the American Society of Echocardiography (ASE), left ventricular diastolic dysfunction can be assessed and graded using measurements obtained from: Pulsed-wave Doppler assessment of mitral inflow, Tissue Doppler imaging of the left ventricular walls, and Left atrial (LA) size (26). Based on left-ventricular diastolic dysfunction as an important pathophysiological mechanism underlying ERAF, echocardiographic parameters such as the E/A ratio, Ea/Aa ratio, E/E’ ratio, pulmonary venous flow, and mitral inflow propagation velocity are commonly used to assess left-ventricular diastolic function (27). Kosiuk et al. (28) demonstrated that an E/A ratio of 1.35 represents the cut-off value with the highest sensitivity and specificity for predicting ERAF. This study also reported that parameters of left ventricular diastolic function did not predict long-term AF recurrence. A recent clinical trial (4) has shown that diastolic function was better in the sinus rhythm compared with the long-standing PerAF recurrence group, with an E/A ratio of 1.5 ± 0.5 vs. 2.2 ± 1.2 (*P* < 0.001) and left-ventricular E/e’ ratio of 8.0 ± 2.1 vs. 10.3 ± 4.1 (*P* < 0.001). Our study retrospectively analyzed the impact of echocardiographic diastolic function indexes, E/A and Ea/Aa, on ERAF. ROC curve analysis showed that the Ea/Aa ratio had superior predictive power for ERAF after RFCA compared with the E/A ratio. Furthermore, E/A and Ea/Aa were treated as categorical variables, and multivariate Cox analysis, adjusted for age, blood pressure level, AF duration, and other factors, showed that only Ea/Aa < 1 significantly increased the risk of ERAF. The E/A ratio is markedly influenced by volume status (e.g., diuretic administration), whereas the Ea/Aa ratio (obtained with tissue Doppler imaging—TDI) more accurately reflects intrinsic myocardial relaxation properties. Thus, we conclude that the Ea/Aa ratio is a more effective predictor of left-ventricular diastolic function than the E/A ratio, which is consistent with the findings from Roten et al. (29). Therefore, for patients undergoing RFCA, it is important to monitor or apply pharmacological interventions for left-ventricular diastolic dysfunction. There is evidence showing that sacubitril valsartan (SV) has a beneficial effect on improving diastolic dysfunction, left-atrial enlargement, and pulmonary hypertension in AF patients (30). SV can reduce AF recurrence after catheter ablation in patients with PerAF at the 1-year follow-up (31). However, it remains to be explored whether SV reduces AF recurrence through improving left-ventricular diastolic function. In this study, we used ultrasound parameters, specifically the Ea/Aa ratio, to indirectly assess left-ventricular diastolic function. The results show that patients with abnormal Ea/Aa values have a significantly higher recurrence rate of AF after catheter ablation.

This provides new evidence for the prognosis of AF ablation in patients with abnormal Ea/Aa values.

PWD and P-wave duration, which can be measured through surface ECG, reflect inhomogeneous atrial depolarization. Prolongation of these parameters may indicate elevated atrial pressure, ischemia, or metabolic stress. These factors, in turn, contribute to atrial structural remodeling, which increases the incidence of AF (32). Li et al. followed up patients who had undergone their first radiofrequency ablation for AF for 1 year. Without conducting early and late recurrence subgroup analyses, the AUC for PWD in predicting AF recurrence was 0.77 (95% CI = 0.56–0.87, *P* < 0.001) (9). Gonna et al. performed electrical cardioversion on patients with PerAF, and 1 month later, PWD was significantly higher in the recurrence group (66 ± 19 ms vs. 57 ± 16 ms, *P* = 0.024). PWD ≥ 62 ms had predictive value for recurrence at 1 month after cardioversion in patients with PerAF (sensitivity 66.7%, specificity 64.6%) (33). Other studies have shown that PWD ≥ 40 ms has strong predictive value for recurrence after radiofrequency ablation for ParAF (34). This study showed that PWD yielded an AUC of 0.92 (95% CI 0.88–0.96) and an optimal cutoff value of 29.5 ms (sensitivity 77.77%, specificity 91.11%). The above study shows that there are differences in the cutoff values of PWD for predicting AF recurrence, which may be related to factors such as the type of AF, the observation period for recurrence, differences in sample size, measurement methods, and errors caused by the personnel performing the measurements.Furthermore, we report a significantly lower PWD threshold compared to previously reported values. This difference is likely attributable to our optimized PWD measurement technique, which utilized QRS-synchronized calibration to reduce respiratory interference.This study offers valuable insights into the prevention and management of ERAF following radiofrequency ablation in patients with atrial fibrillation. For individuals presenting with preoperative Ea/Aa < 1 or PWD ≥ 29.5 ms, tailored perioperative management strategies may be warranted. These could include intensified use of antiarrhythmic therapy prior to ablation and optimization of hemodynamic status, with the aim of minimizing the likelihood of early post-procedural recurrence. During the post-ablation blanking period, enhanced ECG monitoring, such as 24 h Holter monitoring or remote telemetry, is recommended to enable timely detection of ERAF. Importantly, for patients at high risk of ERAF, more stringent lifestyle modifications—including blood pressure control and management of left atrial pressure—should be prioritized. Additionally, these patients may benefit from individualized adjustment of antiarrhythmic drug regimens (e.g., amiodarone or β-blockers), both in terms of dosage and duration, to improve post-ablation outcomes.

## Strength and limitations

The present study had some limitations. First, the sample size was relatively small, and the follow-up period was short, which reduced the analytical power. Second, in the clinical practice, the quality of the collected ECGs may have been suboptimal, and errors could have arisen from differences in measurement methods and the personnel performing the measurements. Third, since this was a retrospective study, the echocardiographic parameters were measured by different individuals, leading to discrepancies in the standards for measuring these parameters, which may explain why continuous variables were not identified as risk factors. Moreover, during the follow-up, there may have been loss to follow-up of patients with asymptomatic recurrence. This study did not include long-term follow-up, so the impact of PWD and Ea/Aa on long-term recurrence after AF radiofrequency ablation remains unclear. Further research and analysis are needed to address these issues.

## Conclusions

To the best of our knowledge, this study is the first attempt to explore the combined predictive value of PWD and Ea/Aa for ERAF after RFCA. The results showed that both PWD and Ea/Aa were independent predictors of ERAF after ablation, with a combined AUC of 0.950, sensitivity of 83.83%, and specificity of 88.88%, which was significantly superior to the prediction using either factor alone. Thus, combining PWD and Ea/Aa testing was found to be a reliable method for predicting ERAF after RFCA. In conclusion, the changes in PWD and Ea/Aa before and after RFCA in AF patients demonstrate preliminary predictive potential for post-ablation early recurrence, and this combined approach could offer valuable clinical guidance for prevention and treatment.

## Data Availability

The original contributions presented in the study are included in the article/Supplementary Material, further inquiries can be directed to the corresponding authors.
