# Supplementary figures and images for: Predictive value of echocardiographic parameter of diastolic dysfunction (Ea/Aa) combined with electrocardiographic P-wave dispersion for the detection of early recurrence of atrial fibrillation after radiofrequency catheter ablation
Source: Front Cardiovasc Med. 2025 Aug 28;12:1585919. doi: 10.3389/fcvm.2025.1585919 (PMC12424079; doi:10.3389/fcvm.2025.1585919)

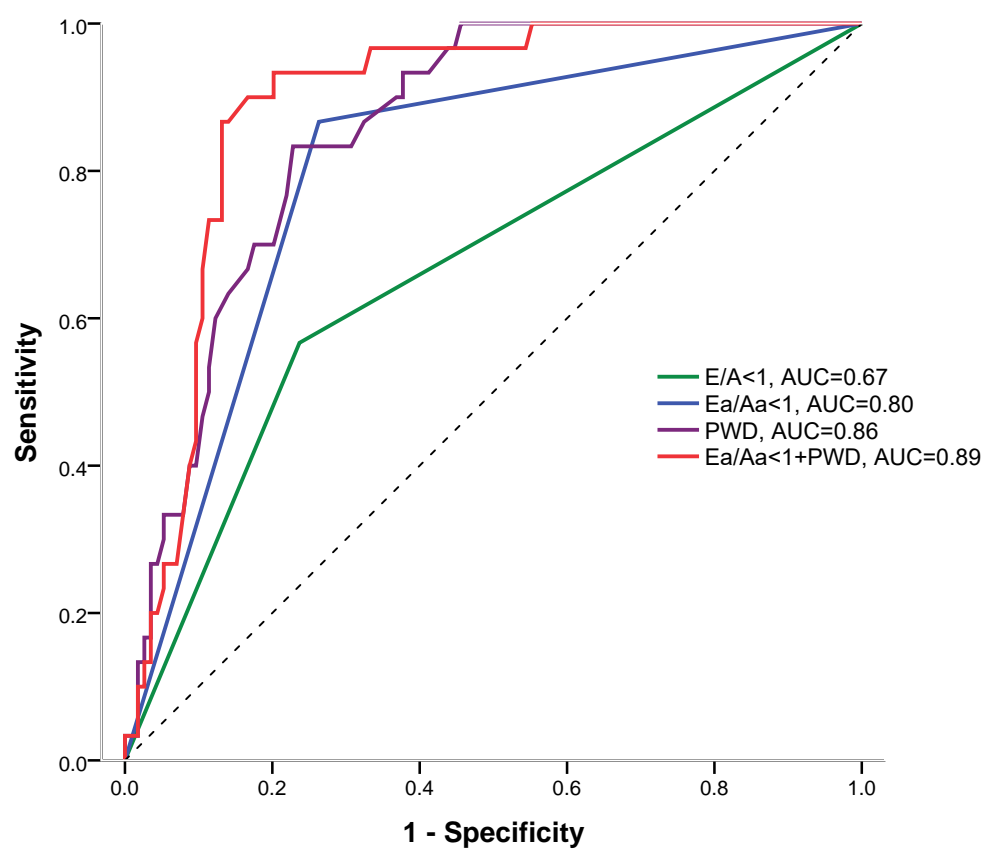

Supplement: Supplementary file 1 [file Datasheet1.pdf]
